# Gastrinome duodénal sporadique

**DOI:** 10.11604/pamj.2015.22.390.6997

**Published:** 2015-12-30

**Authors:** Hicham Sator, Leila Sbihi

**Affiliations:** 1 Hôpital Avicenne, Service de Radiologie Centrale, CHU Ibn Sina, Rabat, Maroc

**Keywords:** Duodénum, gastrinome, sporadique, imagerie, Duodenum, gastrinoma, sporadic, imagery

## Image en medicine

Le gastrinome sporadique duodénal est une tumeur rare, pose souvent des problèmes diagnostiques et thérapeutiques. Il est à l’ origine d'une sécrétion anormale de gastrine entrant dans le cadre du syndrome de Zollinger-Ellison. On rapporte le cas d'un patient âgé de 50 ans, hospitalisé pour l'exploration d'une masse inter-pancréatico-duodénale, de découverte fortuite à l’échographie abdominale. L'interrogatoire avait révélé la notion d’épigastralgies et des épisodes de diarrhée évoluant depuis deux ans. L'examen physique, était sans particularité. Une TDM abdominale avant et après injection de produit de contraste (temps artériel et pancréatique) avait montré une masse bien limitée inter-pancréatico-duodénale, fortement rehaussée après injection. Présentant un signe d’‹‹encorbeillement›› avec la paroi médiale de D2, en faveur d'une origine duodénale de la masse (A, A’). Le complément IRM avait objectivé une lésion duodénale inférieure en discret hypo-signal T1 par rapport au muscle et en hyper signal T2 modéré, relativement homogène, bien limitée, marquant une empreinte sur D2 à droite, sur la tête du pancréas à gauche et sur le pédicule rénal en arrière (B, B’). La fibroscopie oesogastro-duodénale avait objectivé plusieurs ulcères bulbaires et pré pyloriques et a permis la réalisation de multiples biopsies de la lésion permettant de poser le diagnostic de gastrinome duodénal. A postériori, le dosage de la gastrinémie montrait des valeurs élevées à 4 fois lanormale et la scintigraphie nemontrait pas d'autreslocalisations. Une énucléation de la masseétait réalisée et les suites opératoires étaient simples. L'examen anatomopathologique de la pièce opératoire avait confirmé le diagnostic.

**Figure 1:**
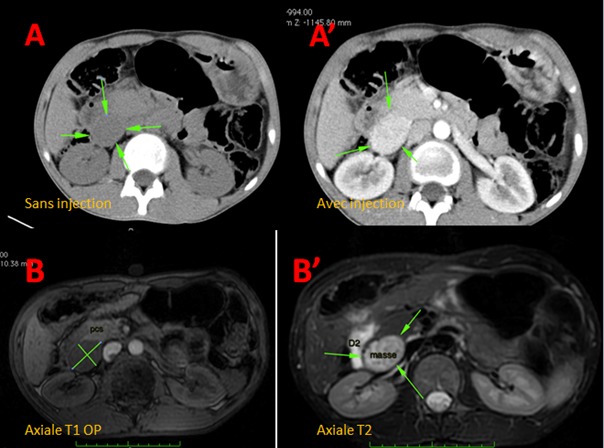
A) TDM abdominale en coupe axiale en contraste spontané: masse interpancréatico-duodénale, bien limitée, présentant le signe d'encorbeillement avec la paroi médiale de D2; A’) TDM abdominale en coupe axiale avec injection de produit de contraste (temps artériel): rehaussement intense et homogène de la masse interpancréatico-duodénale; B) IRM en coupe axiale en pondération T1 OP objectivant une lésion duodénale inférieure, bien limitée, en discret hyposignal par rapport au muscle; B’) IRM en coupe axiale en pondération T2 montrant une lésion et en hypersignal T2 modéré, relativement homogène, bien limitée, marquant une empreinte sur D2 à droite, sur la tête du pancréas à gauche et sur le pédicule rénal en arrière

